# Fate mapping neurons and glia derived from Dbx1‐expressing progenitors in mouse preBötzinger complex

**DOI:** 10.14814/phy2.13300

**Published:** 2017-06-14

**Authors:** Andrew Kottick, Caroline A. Martin, Christopher A. Del Negro

**Affiliations:** ^1^ Department of Applied ScienceThe College of William and MaryWilliamsburgVirginia

**Keywords:** Breathing, central pattern generator, pre‐Bötzinger complex, Respiration

## Abstract

The brainstem preBötzinger complex (preBötC) generates the inspiratory breathing rhythm, and its core rhythmogenic interneurons are derived from Dbx1‐expressing progenitors. To study the neural bases of breathing, tamoxifen‐inducible Cre‐driver mice and Cre‐dependent reporters are used to identify, record, and perturb Dbx1 preBötC neurons. However, the relationship between tamoxifen administration and reporter protein expression in preBötC neurons and glia has not been quantified. To address this problem, we crossed mice that express tamoxifen‐inducible Cre recombinase under the control of the *Dbx1* gene (*Dbx1* ^Cre^ ^ERT^ ^2^) with Cre‐dependent fluorescent reporter mice (*Rosa26* ^tdTomato^), administered tamoxifen at different times during development, and analyzed tdTomato expression in the preBötC of their offspring. We also crossed *Rosa26* ^tdTomato^ reporters with mice that constitutively express Cre driven by *Dbx1* (*Dbx1* ^Cre^) and analyzed tdTomato expression in the preBötC of their offspring for comparison. We show that Dbx1‐expressing progenitors give rise to preBötC neurons and glia. Peak neuronal tdTomato expression occurs when tamoxifen is administered at embryonic day 9.5 (E9.5), whereas tdTomato expression in glia shows no clear relationship with tamoxifen timing. These results can be used to bias reporter protein expression in neurons (or glia). Tamoxifen administration at E9.5 labels 91% of Dbx1‐derived neurons in the preBötC, yet only 48% of Dbx1‐derived glia. By fate mapping Dbx1‐expressing progenitors, this study illustrates the developmental assemblage of Dbx1‐derived cells in preBötC, which can be used to design intersectional Cre/lox experiments that interrogate its cellular composition, structure, and function.

## Introduction

The brainstem preBötzinger Complex (preBötC) generates the rhythm that drives inspiratory breathing movements in mammals (Smith et al. 1991; Feldman et al. 2013; Moore et al. 2013) and its core rhythmogenic interneurons are derived from progenitors that express the transcription factor Dbx1 (i.e., Dbx1 neurons). The putatively rhythmogenic nature of Dbx1 preBötC neurons was first identified using mice that express *LacZ* under the control of the *Dbx1* gene (Pierani et al. 2001). The *LacZ* reporter system was used to quantify peptide receptor localization and transmitter phenotypes of Dbx1 preBötC neurons (Bouvier et al. 2010; Gray et al. 2010). Recently, physiological experiments that address the role of Dbx1 neurons in respiration have employed mice that express constitutive Cre (*Dbx1* ^Cre^; Bielle et al. 2005) or tamoxifen‐inducible Cre (*Dbx1* ^CreERT2^; Hirata et al. 2009), both under the control of the *Dbx1* gene, (Picardo et al. 2013; Wang et al. 2014; Kottick and Del Negro 2015; Revill et al. 2015; Anderson et al. 2016; Cui et al. 2016; Koizumi et al. 2016). However, it is unclear how the timing and dose of tamoxifen administration affects the quantity and proportion of preBötC neurons and glia that express reporter protein, which diminishes the interpretability of these experiments.

Here, we addressed this problem by breeding *Dbx1* ^CreERT2^ females with male Cre‐dependent fluorescent reporter mice (*Rosa26* ^tdTomato^) and administering tamoxifen to pregnant dams at either embryonic day 7.5, 8.5, 9.5, 10.5, or 11.5, which covers the peak window of Dbx1 expression during embryogenesis. We found that Dbx1‐derived progenitors give rise to preBötC neurons and glia, which can be distinguished by molecular markers and morphological criteria. The number of preBötC neurons that expressed tdTomato peaked when tamoxifen was administered at embryonic day 9.5 (E9.5) and was at its nadir at E11.5, while the number of preBötC glia that expressed tdTomato did not depend on tamoxifen administration timing.

These data recap the temporal assemblage of Dbx1‐derived preBötC cells during embryonic development. This information can be applied to bias reporter protein expression in preBötC neurons (or glia) based on tamoxifen administration timing, which optimizes the applicability of *Dbx1* ^CreERT2^ mice for respiratory neurobiology studies.

## Materials and Methods

### Animals

All animal procedures were approved by the institutional animal care and use committee at The College of William and Mary. Female mice that express a tamoxifen‐sensitive Cre recombinase (*Dbx1* ^CreERT2^; CD1 background strain, Hirata et al. 2009) or constitutive Cre recombinase (*Dbx1* ^Cre^; CD1 background strain, Bielle et al. 2005) in cells that express the *Dbx1* gene were crossed with male mice whose *Rosa26* locus was modified to express tdTomato fluorescent protein in a Cre‐dependent manner (*Rosa26* ^tdTomato^; C57BL/6 background strain, stock no. 007905, Jackson Laboratory; Bar Harbor, ME). Female Cre‐driver mice were always crossed with male *Rosa26* ^tdTomato^ mice because CD1 dams yield larger litters (12–16 pups) than C57BL/6 dams (4–10 pups). We did not observe a respiratory phenotype in mixed‐background CD1;C57BL/6 mice breathing normally, but their response to hypoxia may diverge based on disparities of CD1 versus C57BL/6 mice (Zwemer et al., 2007). Males were placed in cages with females at 4:00 pm and removed at 8:00 am the following day for a total of 16‐h breeding time. Tamoxifen (22.5 mg/kg body mass, T5648, Sigma‐Aldrich; St. Louis, MO) was administered to pregnant females via oral gavage at 12:00 pm, either 7, 8, 9, 10, or 11 days after breeding was initiated, depending on the experimental group. Experiments were performed on male and female *Dbx1* ^CreERT2^; *Rosa26* ^tdTomato^ mice aged 6–8 weeks.

### Adeno‐associated virus (AAV) injection

*Dbx1* ^CreERT2^; *Rosa26* ^tdTomato^ mice were anesthetized with intraperitoneal injection of ketamine (100 mg/kg body mass) and aseptic surgeries were performed in a stereotaxic apparatus. The skulls were exposed and unilateral craniotomies (0.5 mm diameter) were performed at 7.0 mm posterior to bregma and 1.3 mm lateral to the midline suture. 200 *μ*L of an AAV that drives GFP expression in neurons via a human synapsin promoter (McLean et al. 2014) (AAV‐hSyn‐GFP, AV‐9‐PV1696, University of Pennsylvania Vector Core; Philadelphia, PA) was injected with a 5‐*μ*L syringe at a depth of 4.7 mm from the dorsal surface of the brain. Incisions were closed with sutures and mice recovered for 4 days before they were killed.

### Transverse medullary slice preparation

Animals were administered a lethal dose of pentobarbital (100 mg/kg body mass) via intraperitoneal injection and then transcardially perfused with 4% paraformaldehyde. Serial transverse brainstem sections (100‐*μ*m thick) were acquired in the rostral to caudal direction using a vibratome and brainstem nuclei were visually analyzed using a bright‐field stereoscope. A single 500‐*μ*m‐thick transverse slice was acquired from each animal at the level of the semi‐compact division of the nucleus ambiguus and the principal loop of the inferior olive, which corresponds to the rostral boundary of the preBötC according to the atlas for neonatal *Dbx1* ^CreERT2^; *Rosa26* ^tdTomato^ reporter mice (Ruangkittisakul et al. 2014).

### Tissue clearing

Tissue preparation and passive clearing were performed as described by Treweek et al. (2015). Transverse brainstem sections were incubated in A4P1 hydrogel solution in a glass vacutainer tube on a rocking platform for 12 h at 4°C. The tube was evacuated of air for 5 min and the hydrogel solution was bubbled with nitrogen gas for an additional 5 min to purge residual oxygen. Tubes containing the brainstem slices and hydrogel solution were incubated in a 37°C water bath for 3 h. Slices were transferred to SDS clearing buffer and incubated at 37°C on an orbital shaker for 8–12 h, or until sufficiently clear. The tissue was washed in 1× PBS on a rotating platform at room temperature for 12 h, followed by a second wash at room temperature for 2 h. Slices were transferred to refractive index matching solution (RIMS) and incubated on an orbital shaker at room temperature for 2 h, then mounted on glass slides in RIMS using 500‐*μ*m ‐thick spacers.

### Immunohistochemistry

Animals were anesthetized and transcardially perfused with 4% paraformaldehyde. Brainstems were postfixed for 12 h at 4°C and sliced to a thickness of 30 *μ*m, and then permeabilized in PBS with 0.4% Triton X‐100 (PBS‐T) for 30 min on a rotating platform at room temperature. Slices were incubated in 10% normal donkey serum (NDS) PBS‐T blocking solution for 1 h on a rotating platform at room temperature. Following blocking, slices were incubated in primary antibody for NeuN (1:1000, MAB377, EMD Millipore; Temecula, CA) or Sox9 (1:500, AF3075, R&D Systems; Minneapolis, MN) diluted in PBS‐T with 2.5% NDS on a rotating platform overnight at 4°C. Three 15‐min washes in PBS were performed, and then slices were incubated in secondary antibody (Donkey anti‐rabbit IgG; 1:400, Abcam ab150073, Cambridge, UK) conjugated to Alexa Fluor 488 in PBS‐T for 2 h on a rotating platform at room temperature. Slices were wet‐mounted onto slides using aqueous, hard mount Vectashield (Vector Laboratories, Burlingame, CA).

### Imaging, morphology analysis, and cell counting

We used a Nikon A1 laser‐scanning confocal microscope (Nikon USA; Melville, NY) to acquire images with a 40x, 1.15 NA water‐immersion objective. FIJI (ImageJ) software (Schindelin et al. 2012) was used to compute the area of cell somata in acquired images. Dbx1‐derived neuron morphology (i.e., soma area) was analyzed in tdTomato^+^/GFP^+^ cells from animals injected with AAV‐hSyn‐GFP. We also analyzed the morphology of tdTomato^+^/NeuN^+^ cells, which resembled tdTomato^+^/GFP^+^ cells in terms of soma area, but the quantified data are from tdTomato^+^/GFP^+^ cells only. We used tdTomato^+^/Sox9^+^ cells to analyze the morphology of Dbx1‐derived glia. Image stacks of the preBötC were acquired within 350 × 350 *μ*m plane at 1‐*μ*m increments in depth. The dorsal limit of the 350 × 350 *μ*m imaging square was placed at the ventral border of the semi‐compact division of the nucleus ambiguus, while the ventral limit of the imaging square was parallel with the dorsal border of the inferior olive. The midline of the 350 × 350 *μ*m square was aligned with the semi‐compact division of the nucleus ambiguus. Image acquisition started at a depth of 100 *μ*m (in the preBötC core) and continued along the z‐axis to a final depth of 200 *μ*m. The preBötC from each slice was imaged unilaterally. For each timed‐tamoxifen condition, neurons and glia were sorted according to morphological criteria (soma area) established using neuron and glia‐specific markers and then counted from confocal image stacks.

### Statistics

We employed resampling statistics (Manly 2007; Motulsky 2014) to quantify the neuronal population that expressed Cre‐dependent fluorophore as a function of tamoxifen delivery during embryonic development in *Dbx1* ^CreERT2^; *Rosa26* ^tdTomato^ mice. In that experiment, we counted the number of Dbx1 preBötC neurons labeled in three separate animals at each date of tamoxifen administration during embryonic development (E7.5, E8.5, E9.5, E10.5, and E11.5, thus 15 animal subjects measured). For each tamoxifen delivery date, we computed the mean number of tdTomato‐labeled neurons. We then tested whether neuronal labeling as a function of tamoxifen administration date was significantly higher (or lower) than what could be explained by chance. First, we disassociated all of the dependent variables (neuron counts) from the value of their corresponding independent variables (date of tamoxifen administration). Then we shuffled the data, randomly assigned neuron counts to a tamoxifen delivery date (sampling from the surrogate data without replacement), and recomputed the sample means. We repeated this shuffling and mean‐computing procedure 10,000 times, assembling a matrix (five tamoxifen dates × 10,000 resampled datasets) of the sample means, from which we extracted the 99% confidence intervals for mean neuron counts of reshuffled data at each tamoxifen administration date. Any sample means from the original dataset that lie outside the 99% confidence intervals for means computed via shuffled and resampled data are statistically significant at alpha = 0.01. We wrote Python scripts to perform resampling statistics according to established algorithms (Manly 2007).

In descriptive statistics, all measurements are reported as mean ± SD. For two‐group experimental designs with abundant samples, where the data adhered to the assumption of being normally distributed, we employed an unpaired *t* test using SSPSS software (IBM, Armonk, NY).

## Results

**Figure 1:**
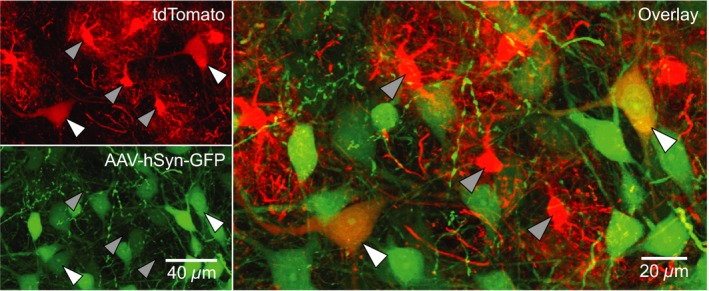
Synapsin promoter‐driven GFP in Dbx1‐derived preBötC cells. Confocal images of *Dbx1* ^Cre^ ^ERT^ ^2^; *Rosa26* ^tdTomato^ mouse preBötC sections 72 h after injection with AAV‐hSyn‐GFP. Dbx1‐derived cells expressed native tdTomato fluorescence. Dbx1‐derived neurons (white arrowheads) co‐expressed tdTomato and GFP. Dbx1‐derived glia (gray arrowheads) only expressed tdTomato.

**Figure 2:**
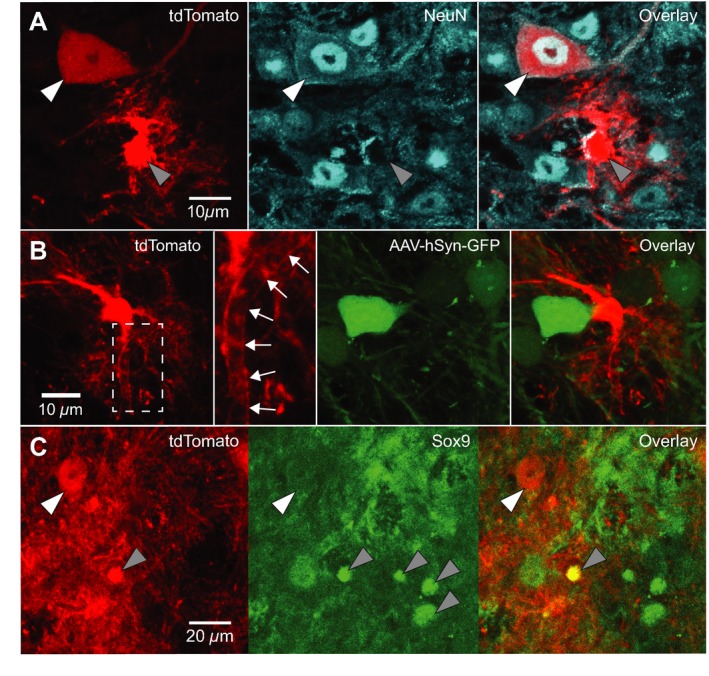
Dbx1‐expressing progenitors give rise to preBötC neurons and glia. A. Confocal images of *Dbx1* ^Cre^ ^ERT^ ^2^; *Rosa26* ^tdTomato^ mouse preBötC sections immunostained for NeuN. Dbx1‐derived neurons (white arrowhead) expressed tdTomato and were immunoreactive for NeuN. Dbx1‐derived glia (gray arrowhead) expressed tdTomato and were not immunoreactive for NeuN. B. Confocal images of *Dbx1* ^Cre^ ^ERT^ ^2^; *Rosa26* ^tdTomato^ mouse preBötC sections 72 h after injection with AAV‐hSyn‐GFP. Dbx1‐derived non‐neuronal cells extend diffuse fibrils that are closely apposed to the outer surface of microvasculature (white arrows). C, Confocal images of *Dbx1* ^Cre^ ^ERT^ ^2^; *Rosa26* ^tdTomato^ mouse preBötC sections immunostained for Sox9. Glia (gray arrowheads) were immunoreactive for Sox9. Dbx1‐derived neurons (white arrowhead) expressed tdTomato but were not immunoreactive for Sox9. Dbx1‐derived glia expressed tdTomato and were immunoreactive for Sox9 (see “overlay” panel).

First, to characterize the morphological properties of Dbx1‐derived preBötC cells, we crossed *Dbx1* ^CreERT2^ mice with *Rosa26* ^tdTomato^ mice, administered tamoxifen to pregnant dams at E10.5, and screened their offspring (aged 6–8 weeks) for neuronal and glial markers. Three days after injecting *Dbx1* ^CreERT2^; *Rosa26* ^tdTomato^ mice with AAV‐hSyn‐GFP, we identified Dbx1‐derived neurons based on cytosolic tdTomato and GFP expression (*n* = 37, Fig. 1, tdTomato^+^/GFP^+^ cells, white arrowheads). Alternatively, in *Dbx1* ^CreERT2^; *Rosa26* ^tdTomato^ mice not injected with AAV‐hSyn‐GFP, we identified Dbx1‐derived neurons based on cytosolic tdTomato expression and nuclear NeuN immunoreactivity (*n* = 17 Fig. 2A, tdTomato^+^/NeuN^+^ cells, white arrowheads). In contrast, cells exhibiting diffuse fibrils that were closely apposed to the outer surface of microvasculature (Fig. 2B and 2B inset, tdTomato^+^ cell), which did not express GFP driven by synapsin promoter (Fig. 1, gray arrowheads) and were not immunoreactive for NeuN (Fig. 2A, gray arrowhead), were deemed to be glia. These Dbx1‐derived glia were immunoreactive for the astrocyte marker Sox9 in some but not all cases (*n* = 34 of 110 glia examined, Fig. 2C, gray arrowheads).

**Figure 3:**
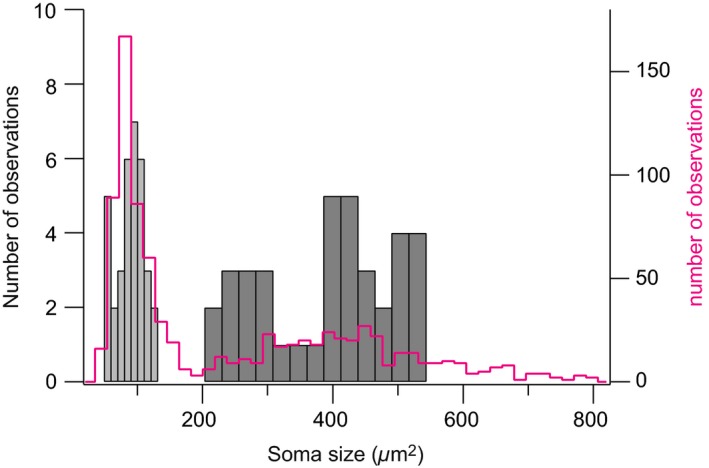
Distribution of soma size (area) of Dbx1‐derived preBötC neurons and glia. The histogram for neurons is plotted in dark gray bars (right, bin size 26 *μ*m^2^). The histograms for glia are plotted in light gray bars (left, bin size 10 *μ*m^2^). Statistics (mean ± SD) are given in the main text. The magenta line represents the soma size (area) distribution of all cells in the timed‐tamoxifen experiments (*n* = 1,049 cells counted in 18 different animals, bin size 18 *μ*m^2^).

We measured the soma area of Dbx1‐derived tdTomato^+^/GFP^+^ neurons as well as tdTomato^+^/Sox9^+^ glia and plotted the distributions, which did not overlap (Fig. 3). The somata of neurons (area = 389 ± 102 *μ*m^2^, *n* = 37) were larger than those of glia (area = 88 ± 22 *μ*m^2^, *n* = 34, unpaired *t* test, *P* < 0.0001). Neurons had fewer primary processes than glia (3–5 in neurons compared to 6–8 in glia), but their neuronal processes were notably thicker (diameter of ~1 *μ*m in neurons vs. ~200 nm in glia). The number and diameter of primary processes measured here in juvenile Dbx1 preBötC neurons match those of preBötC interneurons studied perinatally. The round somata and the highly branched fibrils we observed in preBötC glia are characteristic of protoplasmic astrocytes (Stolt et al. 2003; Rowitch and Kriegstein 2010; Robel et al. 2011).

**Figure 4:**
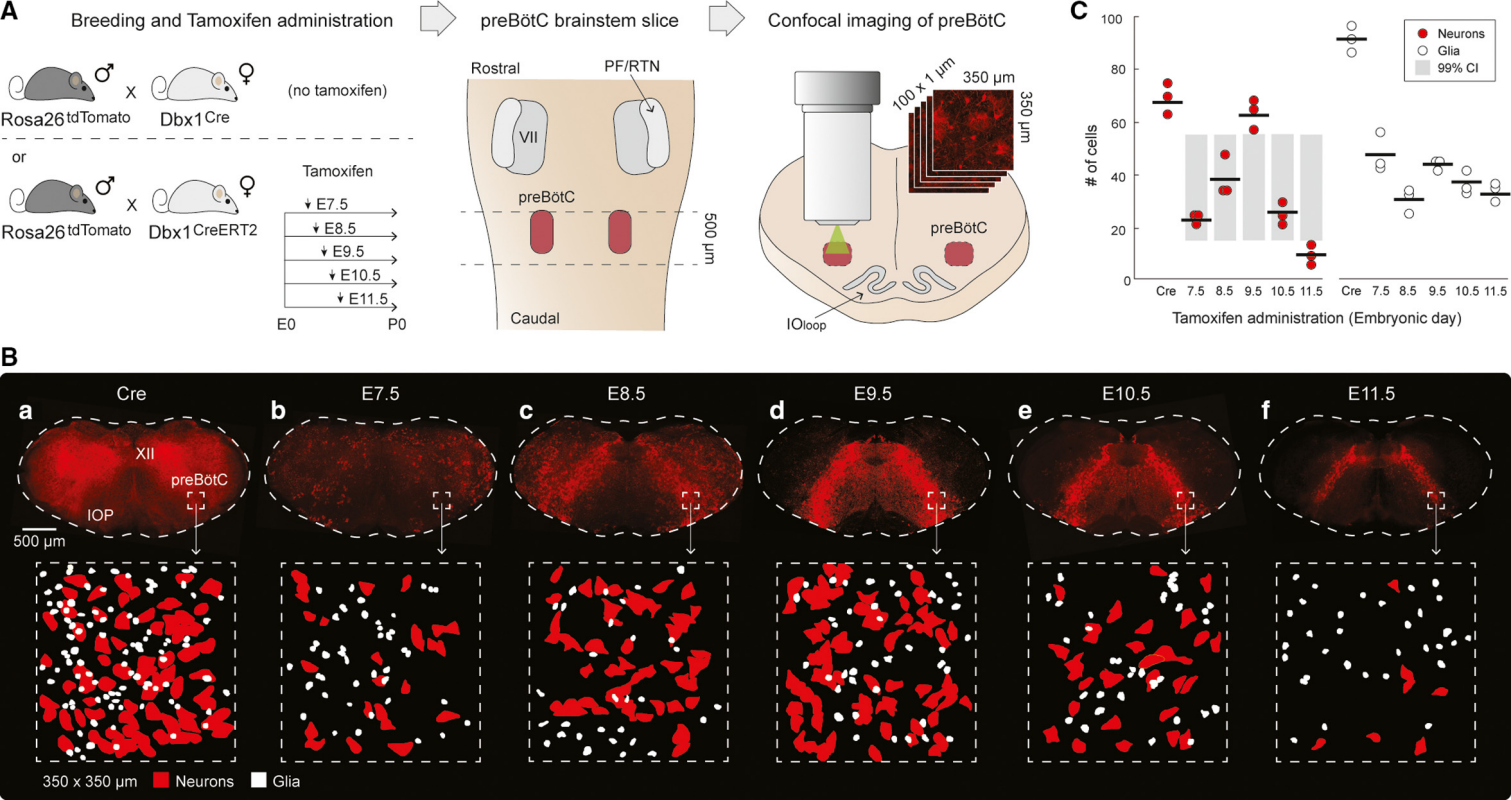
Relationship between tamoxifen timing and tdTomato expression. A. Experimental setup: *Rosa26* ^tdTomato^ mice were crossed with either *Dbx1* ^Cre^ or *Dbx1* ^Cre^ ^ERT^ ^2^ mice. Pregnant *Dbx1* ^Cre^ ^ERT^ ^2^ dams received tamoxifen at either embryonic day 7.5, 8.5, 9.5, 10.5, or 11.5 (E7.5‐11.5). 500‐*μ*m‐thick brainstem sections encompassing the preBötC were optically cleared and unilateral confocal sections of the preBötC (350 × 350 *μ*m; 100 × 1 *μ*m in Z) were acquired. B. tdTomato expression in cleared medullary preBötC sections from *Dbx1* ^Cre^; *Rosa26* ^tdTomato^ (marked ‘Cre’) and *Dbx1* ^Cre^ ^ERT^ ^2^; *Rosa26* ^tdTomato^ (marked ‘E7.5‐11.5’) mice (top row) and masks of neurons (red) and glia (white) from the preBötC test volume of 350 × 350 × 100 *μ*m (bottom row). C. The mean number of Dbx1 preBötC neurons (red circles) and glia (white circles) counted in the preBötC test volume of 350 × 350 × 100 *μ*m for each animal. Means are indicated by horizontal lines. Gray bars reflect the 99% confidence intervals for shuffled and resampled data. The mean number of neurons labeled following tamoxifen at E9.5 and E11.5 are statistically significant (*P* < 0.01).

Next, we aimed to identify the relationship between tamoxifen administration and tdTomato expression. We crossed *Dbx1* ^CreERT2^ females with *Rosa26* ^tdTomato^ males and administered tamoxifen via oral gavage to pregnant dams at embryonic days 7.5, 8.5, 9.5, 10.5, or 11.5 (E 7.5–11.5; Fig. 4A). For comparison, we crossed constitutive *Dbx1* ^Cre^ females with *Rosa26* ^tdTomato^ males, whose offspring express tdTomato in all Dbx1‐derived cells. We performed passive clearing on 500‐*μ*m‐thick transverse brainstem slices from 6‐ to 8‐week‐old offspring, and acquired confocal images (350 × 350 *μ*m) of the preBötC from a 100–*μ*m ‐thick section of the preBötC (Fig. 4A).

TdTomato expression was widespread in *Dbx1* ^Cre^; *Rosa26* ^tdTomato^ sections. Labeled somata were densest in the dorsal region of the brainstem, lateral to the hypoglossal motor nucleus, and in wide (400‐500 *μ*m) bands extending dorsomedially from the hypoglossal motor nucleus to the ventrolateral edge of the brainstem (Fig. 4 Ba, top). Although hypoglossal motor neurons did not express tdTomato, neuropil labeling occurred within the hypoglossal motor nucleus, as previously shown using *Dbx1* ^CreERT2^ mice (Ruangkittisakul et al. 2014). The morphological criteria established in Figs. 1, 2, 3 suggest that cell soma area of 200 *μ*m^2^ differentiates neurons (area > 200 *μ*m^2^) from glia (area < 200 *μ*m^2^). The distribution of soma area for all tdTomato‐labeled cells (*n* = 1049 cells counted in 18 different animals) is bimodal with a break at 190 *μ*m^2^, consistent with two populations of cells: Dbx1‐derived neurons and glia, which are differentiable by size. In *Dbx1* ^Cre^; *Rosa26* ^tdTomato^ mice, we identified and counted 70 ± 1 tdTomato‐labeled neurons and 92 ± 4 tdTomato‐labeled glia within the designated 350 × 350 × 100 *μ*m imaging volume from the core of the preBötC (*n* = 3 cleared slices; Fig. 4B,C).

In *Dbx1* ^CreERT2^; *Rosa26* ^tdTomato^ offspring, when tamoxifen was administered to pregnant dams at E7.5, tdTomato expression was diffuse and occurred mostly in somata dorsal and lateral to the hypoglossal motor nucleus (Fig. 4Bb). In the preBötC, we counted 23 ± 1 tdTomato‐labeled neurons and 47 ± 7 tdTomato‐labeled glia, which reflects 34% and 52% of the total number Dbx1‐derived neurons and glia found, respectively, in the *Dbx1* ^Cre^; *Rosa26* ^tdTomato^ mouse tissue (*n* = 3 cleared slices; Fig. 4B,C).

In addition to diffuse labeling in dorsal and lateral brainstem regions, an inverted U‐shaped expression pattern that was anchored at the ventrolateral edge of the brainstem and extended dorsomedially to the hypoglossal motor nucleus became visible when tamoxifen was administered to pregnant dams at E8.5 (Fig. 4Bc). We counted 38 ± 7 tdTomato‐labeled neurons and 30 ± 6 tdTomato‐labeled glia, which reflects 55% and 34% of the total number of Dbx1‐derived neurons and glia found, respectively, in the *Dbx1* ^Cre^; *Rosa26* ^tdTomato^ mouse tissue (*n* = 3 cleared slices; Fig. 4B,C).

When tamoxifen was administered to pregnant dams at E9.5, the majority of tdTomato‐labeled somata were confined to bands extending from the hypoglossal motor nucleus to the ventrolateral edges of the brainstem (Fig. 4Bd). Dense neuropil labeling was visible in the hypoglossal motor nucleus (also see Fig. 7 in Ruangkittisakul et al. 2014) similar to the offspring of *Dbx1* ^Cre^ mice. We counted 63 ± 4 tdTomato‐labeled neurons and 42 ± 1 tdTomato‐labeled glia in the preBötC, which reflects 91% and 48% of the total number of Dbx1‐derived neurons and glia found, respectively, in the *Dbx1* ^Cre^; *Rosa26* ^tdTomato^ mouse tissue (*n* = 3 cleared slices; Fig. 4B,C).

When tamoxifen was administered to pregnant dams at E10.5, tdTomato‐labeled somata were more tightly confined to ~200‐*μ*m wide bands extending dorsomedially from the hypoglossal motor nucleus to the ventrolateral edges of the brainstem (Fig. 4Be). Labeling of neuropil in the medial region of the slice, between the U‐shaped bands, as well as in the hypoglossal motor nucleus, dwindled compared to the offspring of dams who received tamoxifen at E9.5. We counted 26 ± 3 tdTomato‐labeled neurons and 38 ± 3 tdTomato‐labeled glia in the preBötC, which reflects 37% and 43% of the total number of Dbx1‐derived neurons and glia found, respectively, in the *Dbx1* ^Cre^; *Rosa26* ^tdTomato^ mouse tissue (*n* = 3 cleared slices; Fig 4B,C).

When tamoxifen was administered at E11.5, tdTomato‐labeled somata were tightly confined to bands extending from the hypoglossal motor nucleus and terminating in the preBötC (Fig. 4Bf). The bands were less prominent than in the offspring of dams who received tamoxifen between E8.5 and E10.5, and did not reach the ventral border. Neuropil was sparsely labeled in the hypoglossal motor nucleus. We counted 9 ± 3 tdTomato‐labeled neurons and 34 ± 3 tdTomato‐labeled glia, which reflects 14% and 38% of the total number of Dbx1‐derived neurons and glia found, respectively, in the *Dbx1* ^Cre^; *Rosa26* ^tdTomato^ mouse tissue (*n* = 3 cleared slices; Fig 4B,C).

The number of tdTomato‐labeled Dbx1 preBötC neurons peaked for tamoxifen administration at E9.5. That peak was statistically significant (alpha = 0.01) because it exceeded the upper limit of the 99% confidence intervals for the shuffled and resampled data (see Methods). Conversely, the nadir of tdTomato‐labeled Dbx1 preBötC neurons was associated with tamoxifen delivery at E11.5, which was also statistically significant (alpha = 0.01) by a similar argument, that is, the actual mean was below the lower limit of the 99% confidence intervals from the resampled surrogate data (Manly 2007; Motulsky 2014).

## Discussion

Here, we describe how tamoxifen administration timing in pregnant *Dbx1* ^CreERT2^ mice, crossed with an appropriate Cre‐responder line, influences reporter protein expression in the preBötC of their offspring. Using homozygous *Dbx1* ^CreERT2^ mice, we show that Dbx1‐derived progenitors give rise to morphologically distinct preBötC neurons and glia. Furthermore, we fate‐map Dbx1‐expressing progenitors by administering tamoxifen at different times during development, then quantify their progeny (as either neurons or glia) in the preBötC of juvenile mice. These data demonstrate the total number, as well as the relative proportion, of Dbx1‐derived preBötC neurons and glia that express reporter protein as a function of the timing of tamoxifen administration during embryogenesis. Neuronal reporter expression peaks when tamoxifen is administered at E9.5 and is at its low point when tamoxifen is administered at E11.5, but there is no relationship between tamoxifen timing and glial reporter expression.

We observed tdTomato expression in Dbx1‐derived cells, which included both neurons and glia differentiated by molecular markers. tdTomato‐expressing cells that did not express GFP driven by a synapsin promoter and were not immunoreactive for NeuN, yet were immunoreactive for Sox9, were deemed glia. Dbx1‐derived glia measured smaller in soma area, with thinner and more densely branched processes. tdTomato‐expressing cells that always expressed GFP driven by a synapsin promoter and were immunoreactive for NeuN were deemed neurons. Dbx1‐derived neurons were larger in soma area (measured from GFP‐expressing cells), with thicker primary processes (i.e., dendrites). These data are consistent with Bouvier et al. (2010) and Gray et al. (2010), who also identified Dbx1‐derived glia as well as Dbx1‐derived neurons in the preBötC, and indicate that hindbrain Dbx1 proliferative zones generate preBötC neurons and glia that can be reliably distinguished morphologically.

Classifying glia is challenging because there are several subtypes, for which few (if any) subtype‐specific molecular markers are currently available. Nearly one‐third of Dbx1‐derived preBötC glia were immunoreactive for Sox9, which suggests that they might be protoplasmic astrocytes. That identity would be consistent with Gray et al. (2010), who showed that a subset of Dbx1‐derived preBötC cells are immunoreactive for the astrocyte marker S100*β*. Notwithstanding, Sox9 and S100*β* have also been implicated in oligodendrocyte development (Stolt et al. 2003), and not all astrocytes express either marker. Oligodendrocytes are morphologically diverse and in some circumstances resemble the Dbx1‐derived preBötC glia (e.g., compare our Fig. 2A,B to Fig. 3G in Zhao et al. 2016). Furthermore, Dbx1‐expressing precursors of the ventricular zone give rise to a subpopulation of oligodendrocytes in the dorsal spinal cord (Fogarty et al. 2005), so it would not be surprising that Dbx1 gives rise to oligodendrocytes in the hindbrain too. Dbx1‐derived preBötC glia could include both astrocytes and oligodendrocytes.

Why is it significant that Dbx1‐derived preBötC glia express reporter protein? Studies of respiratory rhythmogenesis typically focus on neuronal mechanisms. However, astrocytes are chemosensitive and release ATP in response to hypoxia‐induced intracellular Ca^2+^ elevations (Angelova et al. 2015; Turovsky et al. 2016). As a result, medullary astrocytes have been implicated in central chemoreception and respiratory control (Gourine et al. 2005; Funk et al. 2015). Further, at least one report shows preBötC astrocytes that exhibit rhythmic Ca^2+^ elevations preceding inspiratory neuronal activity, and whose transient activation elicits single or burst firing of action potentials in inspiratory neurons (Okada et al. 2012). Because Dbx1‐derived preBötC glia might influence respiratory rhythm, their physiological impact in experiments that utilize Dbx1‐Cre‐driver mice must be considered. For example, Cre‐driver mice crossed with Cre‐dependent channelrhodopsin reporters enable photo‐activation of Dbx1 preBötC neurons (Kottick and Del Negro 2015; Anderson et al. 2016; Cui et al. 2016). It is reasonable to assume that Dbx1‐derived glia also express channelrhodopsin, and were photo‐activated in parallel with Dbx1 neurons in those studies. Similarly, Cre‐driver mice have been crossed with Cre‐dependent archaerhodopsin reporters to transiently suppress Dbx1 preBötC neurons. (Koizumi et al. 2016; Vann et al. 2016). In some systems, mastication for example, astrocytes appear to play an integral role in rhythm and burst generation (Morquette et al. 2015). However, for respiratory rhythm in the preBötC, the role of astrocytes is far from clear at this stage.

Although transient activation or suppression of preBötC glia might impact the results of physiological experiments, our data suggest that any confounding influence of reporter‐expressing glia can be strategically mitigated via intersectional mouse genetics. Specifically, breeding and administering tamoxifen can bias reporter protein expression toward neurons and minimize reporter protein expression in glia. When tamoxifen is administered at E9.5, *Dbx1* ^CreERT2^; *Rosa26* ^tdTomato^ mice express tdTomato in 91% of the total neurons that express tdTomato in *Dbx1* ^Cre^; *Rosa26* ^tdTomato^ mice, but only 48% as many glia. Further, the density of tdTomato‐expressing neuropil in the preBötC of *Dbx1* ^CreERT2^; *Rosa26* ^tdTomato^ mice is lower than in their *Dbx1* ^Cre^; *Rosa26* ^tdTomato^ counterparts. This facilitates identification of Dbx1‐derived neuronal somata based on morphology, particularly soma area, with a reasonable cutoff at 200 *μ*m^2^ for neonatal mice (Fig. 3). To minimize the influence of glia in physiological experiments aimed at manipulating the activity of Dbx1 neurons, we recommend using *Dbx1* ^CreERT2^ mice (administering tamoxifen at E9.5) as opposed to *Dbx1* ^Cre^ mice. We also recommend using *Dbx1* ^CreERT2^ mice for targeted recordings or ablations, because their low background fluorescence facilitates visual identification and differentiation of Dbx1 cells (i.e., neurons or glia).

Because Cre‐mediated recombination does not occur in all Dbx1‐derived cells when using *Dbx1* ^CreERT2^ mice, we recommend using constitutive *Dbx1* ^Cre^ mice for experiments that cannot tolerate false negatives, that is, cells that do not express reporter protein, but are nonetheless Dbx1‐derived. For example, *Dbx1* ^Cre^ mice are preferable for experiments that involve electrophysiological, RT‐PCR, or RNA‐Seq comparisons of Dbx1‐ and non‐Dbx1‐derived preBötC cells, or any experiment that involves identifying non‐Dbx1 cells based on lack of reporter protein expression.

These data can also be used to devise strategies to address the role of Dbx1‐derived glia in respiratory rhythm or pattern generation. Crossing *Dbx1* ^CreERT2^; *Rosa26* ^tdTomato^ mice and administering tamoxifen at E11.5 sparsely labels neuropil in the preBötC and yet labels far more glia than neurons. Under these circumstances, detailed morphologies of glia could be acquired in images that remain relatively uncluttered by fluorescent Dbx1‐derived neurons, and glia would be easy to distinguish for targeted electrophysiological recordings or calcium imaging. One could ablate Dbx1‐derived astrocytes by crossing constitutive *Dbx1* ^Cre^ mice with Aldh1L1‐eGFP‐Stop‐DTA mice (Tsai et al. 2012; stock no. 026033, Jackson Laboratory), which would activate Diphtheria toxin expression in (and subsequently destroy) Aldh1 l1‐expressing Dbx1‐derived astrocytes. However, this experiment would destroy all Dbx1‐derived Aldh1 l1‐expressing cells, including those outside of the preBötC. We are not currently aware of any available transgenic strains that would permit targeted manipulation of Dbx1‐derived preBötC glia, but it might involve crossing Dbx1‐Cre‐driver mice with Cre‐dependent reporters whose genomes have been modified to express a light gated ion channel or proton pump under the control of a glia‐specific promoter (and then light could be selectively delivered to the preBötC).

Another viable approach to selectively manipulate Dbx1‐derived preBötC neurons could employ a double‐stop system featuring Cre‐ and FlpO‐ recombinase‐dependent reporters (Britz et al. 2015). For example, a transgenic line containing two stop codons flanked by LoxP and FRT sites, followed by a reporter transgene could be crossed with a Dbx1 Cre driver. Cre recombinase would remove the first stop codon in Dbx1‐derived cells, and a synapsin‐dependent FlpO vector could be injected into the preBötC of postnatal mice to eliminate the FRT‐flanked stop codon. This double‐stop approach would target in reporter protein expression only to Dbx1‐derived preBötC neurons.

Dbx1 preBötC neurons have respiratory rhythm‐generating and premotor function (Bouvier et al. 2010; Gray et al. 2010; Picardo et al. 2013; Wang et al. 2014; Cui et al. 2016; Koizumi et al. 2016; Vann et al. 2016), and represent a potential therapeutic target for apnea of prematurity, obstructive sleep apnea, and respiratory failure in geriatrics or neurodegenerative disease. Therefore, it is physiologically relevant to determine the anatomical boundaries of the preBötC and quantify its constituent interneurons.

Studies of rhythmically active brainstem preparations suggest that the preBötC extends along the rostral‐caudal axis for ~150 *μ*m in neonatal mice (Ruangkittisakul et al. 2011, 2014) and ~200 *μ*m in neonatal rats (Ruangkittisakul et al. 2006, 2008), where the preBötC core is centered at the level of the semicompact nucleus ambiguus, the principal loop of the inferior olive is fully developed, and the medial inferior olive shows a sharp dorsomedial stalk‐like structure. Histological analysis of these landmarks in 3‐month‐old mice suggests that the preBötC spans 440 *μ*m in the rostral‐caudal axis in juveniles (similar to the mice used in this study) (Franklin and Paxinos 2013). In the transverse plane, the dorsal border of the preBötC is located ventral to the nucleus ambiguus and its ventral border is parallel to the dorsal boundary of the inferior olive (Gray et al. 2001; Ruangkittisakul et al. 2006, 2008, 2011, 2014), a region spanning roughly 350 × 350 *μ*m in our brainstem sections (Fig. 4). Therefore, we estimate the preBötC occupies a volume of 350 × 350 × 440 *μ*m in our *Dbx1* ^Cre^; *Rosa26* ^tdTomato^ mice. We identified an average of 63 neurons in a 350 × 350 × 100 *μ*m region in the center of the preBötC. Extrapolating these data for the estimated dimensions of the preBötC (i.e., 440 *μ*m in the anterior‐posterior axis) provides an estimate of ~554 Dbx1‐derived preBötC neurons. This enumeration matches other estimates of the essential preBötC core such as ~550 neurons in neonatal Dbx1 reporter mice (Wang et al. 2014) and ~600 neurons in adult rats (Gray et al. 2001). We therefore conclude that 550‐600 neurons is a reasonable estimate for the size of the rhythmogenic neuronal core of the preBötC in mice.

Dbx1‐derived preBötC neurons are born at E10.5 (Bouvier et al. 2010); yet, we report that optimal neuronal labeling occurs when tamoxifen is administered at E9.5, which may appear to contradict the prior reports. However, BrdU, which is administered via intraperitoneal injection for birth dating in *Dbx1* ^LacZ^ knock‐in mice, takes effect almost immediately, whereas tamoxifen (which is administered via oral gavage for CreERT2‐dependent reporter labeling) has a lag time of approximately 24‐h due to digestion, Cre‐activation, as well as transcription and translation of reporter proteins. Given the inherent delays associated with tamoxifen‐triggered Cre‐recombination and reporter expression, the BrdU and fluorescent reporter data are in fact congruent. Recognizing that difference, Gray et al. (2010), who used the same *Dbx1* ^CreERT2^ mouse, also reported that tamoxifen administration at E9.5 labels the majority of rhythmogenic preBötC neurons (Gray et al. 2010).

The precise kinetics of CreERT2 nuclear translocation and subsequent cleavage of LoxP sites following tamoxifen administration is not well‐defined and difficult to assess. Here, we show that the number of tdTomato‐expressing neurons and glia counted for each tamoxifen administration time point (E7.5‐11.5) sums to greater than 100% of the total number of Dbx1‐derived neurons and glia (counted in experiments using the constitutive Cre mouse). That disparity can be explained by CreERT2 nuclear activity that persists for more than 24 h (Reinert et al. 2012).

This study describes the developmental assemblage of the preBötC, both its neuronal and glial components derived from Dbx1‐expressing progenitors. These findings can be used to bias reporter protein expression toward Dbx1 preBötC neurons or could be applied to investigate the respiratory role(s) of Dbx1‐derived glia. By optimizing the use of Cre‐driver mice coupled with Cre‐dependent reporters, one can design better experiments to interrogate the cellular mechanisms underlying respiratory rhythmogenesis and pattern formation.

## Conflict of Interest

The authors declare no competing financial interests.
